# Ruminal Degradation of Rumen-Protected Glucose Influences the Ruminal Microbiota and Metabolites in Early-Lactation Dairy Cows

**DOI:** 10.1128/AEM.01908-20

**Published:** 2021-01-04

**Authors:** Yapin Wang, Xuemei Nan, Yiguang Zhao, Yue Wang, Linshu Jiang, Benhai Xiong

**Affiliations:** a State Key Laboratory of Animal Nutrition, Institute of Animal Science, Chinese Academy of Agricultural Sciences, Beijing, China; b Beijing Key Laboratory for Dairy Cow Nutrition, Beijing University of Agriculture, Beijing, China; University of Helsinki

**Keywords:** rumen-protected glucose, negative energy balance, 16S rRNA, metabolomics, microbiota, early lactation

## Abstract

Rumen-protected glucose (RPG) plays an important role in alleviating the negative energy balance of dairy cows. This study used a combination of rumen microbes 16S and metabolomics to elucidate the changes of rumen microbial composition and rumen metabolites of different doses of RPG’s rumen degradation part in early-lactation dairy cows. Twenty-four multiparous Holstein cows in early lactation were randomly allocated to control (CON), low-RPG (LRPG), medium-RPG (MRPG), or high-RPG (HRPG) groups in a randomized block design. The cows were fed a basal total mixed ration diet with 0, 200, 350, and 500 g of RPG per cow per day, respectively. Rumen fluid samples were analyzed using Illumina MiSeq sequencing and ultrahigh-performance liquid chromatography coupled to quadrupole time-of-flight mass spectrometry. MRPG supplementation increased bacterial richness and diversity, including increasing the relative abundance of cellulolytic bacteria, such as *Ruminococcus*, *Lachnospiraceae_NK3A20_group*, *Ruminiclostridium*, and *Lachnospiraceae_UCG-008*. MRPG significantly increased the concentrations of acetate, propionate, butyrate, and total volatile fatty acid in the rumen. Ruminal fluid metabolomics analysis showed that RPG supplementation could significantly regulate the synthesis of amino acids digested by protozoa in the rumen. Correlation analysis of the ruminal microbiome and metabolome revealed some potential relationships between major bacterial abundance and metabolite concentrations. Our analysis found that RPG supplementation of different doses can change the diversity of microorganisms in the rumen and affect the rumen fermentation pattern and microbial metabolism and that a daily supplement of 350 g of RPG might be the ideal dose.

**IMPORTANCE** Dairy cows in early lactation are prone to a negative energy balance because their dry matter intake cannot meet the energy requirements of lactation. Rumen-protected glucose is used as an effective feed additive to alleviate the negative energy balance of dairy cows in early lactation. However, one thing that is overlooked is that people often think that rumen-protected glucose is not degraded in the rumen, thus ignoring its impact on the microorganisms in the rumen environment. Our investigation and previous experiments have found that rumen-protected glucose is partially degraded in the rumen. However, there are few reports on this subject. Therefore, we conducted research on this problem and found that rumen-protected glucose supplementation at 350 g/day can promote the development and metabolism of rumen flora. This provides a theoretical basis for the extensive application of rumen bypass glucose at a later stage.

## Abstract (precis)

Dairy cows in early lactation are prone to a negative energy balance because their dry matter intake cannot meet the energy requirements of lactation. Rumen-protected glucose is used as an effective feed additive to alleviate the negative energy balance of dairy cows in early lactation. However, one thing that is overlooked is that people often think that rumen-protected glucose is not degraded in the rumen, thus ignoring its impact on the microorganisms in the rumen environment. Our investigation and previous experiments have found that rumen-protected glucose is partially degraded in the rumen. However, there are few reports on this subject. Therefore, we conducted research on this problem and found that rumen-protected glucose supplementation at 350 g/day can promote the development and metabolism of rumen flora. This provides a theoretical basis for the extensive application of rumen bypass glucose at a later stage.

## INTRODUCTION

Cows in early lactation are likely to be in negative energy balance (NEB) due to inadequate feed intake, causing fatty liver, ketosis, and other metabolic diseases (1). Manipulating the rumen microbiome and meeting global livestock challenges through the introduction of dietary interventions during early life have recently emerged as promising new technologies (2). Glucose is a key nutrient for the maintenance of basic functions of body tissues and the synthesis of milk during lactation (3). Rumen-protected glucose (RPG) improved the lactation performance and enhanced the immune function of dairy cows in early lactation (4, 5). However, Li et al. (4) reported that a supplement amount of RPG of 200 g/day during the transition period was not sufficient to meet the increased requirement in energy consumption and milk production and therefore more body fat would be mobilized to meet the energy demand, which would have a negative impact on hepatic function. In addition, Wang et al. (6) measured the rumen degradation rate of RPG and demonstrated that different doses of RPG had a certain influence on rumen fermentation indexes of dairy cows, which indicated that RPG does not pass through the rumen completely and has an influence on rumen fermentation, but these aspects are often ignored. Therefore, we studied here the effects of RPG in rumen degradation on the function and metabolic properties of the rumen microbiota of dairy cows.

The rumen is a complex ecosystem consisting of anaerobic bacteria, fungi, archaea, and protozoa (2). Bacteria are the main microorganisms in the rumen (>91% of the whole microbiome) which produce volatile fatty acids (VFA) and microbial proteins that provide cows with more than 70% of the required energy and 60% of the nonamino nitrogen (7–9). However, microorganisms also use various metabolites with complex activities in the rumen for their own reproduction, and approximately 55 to 60% of the metabolites in rumen fluid were associated with the rumen microbiota (10). Rumen microbes degrade carbohydrates through fermentation to generate energy for microbial growth, and the final product of microbes acts as an energy substrate, inflammation regulator, and signaling molecule (10, 11). Therefore, the role of the rumen microbiome in host health goes far beyond the local role of the rumen. Previous studies have revealed the interaction between the diversity or unique niche of functional categories and metabolic factors that control microbial metabolism and related nutrient formation pathways (12, 13). Accumulating evidence indicated that rumen metabolic disorders related to changes in the rumen microbiota composition are important risk factors for disease development (14, 15). Furthermore, it has been shown that feeding strategies and dietary changes significantly affected rumen microbiome (16, 17). A stable microbial community is necessary to maintain ruminant health. Therefore, a better understanding of the composition of rumen microbial communities and the interaction between the microbiome and the metabolome is essential to understand the impact of RPG on rumen microbiota function.

To the best of our knowledge, no previous study has investigated the effects of RPG’s rumen degradation part on rumen microbiota and metabolism using integrative analysis of metabolome and microbiome. Amplicon sequencing of 16S rRNA in bacteria remains the most cost-effective and convenient tool, providing valuable phylogenetic information for comparing microbial diversity in a large number of environmental samples (18). Metabolomics has been used to characterize the metabolism of rumen fluid in dairy cows (19). Untargeted metabolomics by liquid chromatography-mass spectrometry (LC-MS) is used to screen the differential metabolites in the rumen among different treatment groups of dairy cows (20). Here, 16S rRNA sequencing on an Illumina MiSeq platform and ultrahigh-performance liquid chromatography coupled to quadrupole time-of-flight mass spectrometry (UPLC-QTOF/MS) were applied to characterize ruminal profiles of microbiota and metabolites and to uncover the interaction between them.

## RESULTS

### Rumen fermentation parameters.

The proportions of acetate, propionate, butyrate, and total volatile fatty acid (TVFA) in the MRPG group were the highest among all groups, and they were all significantly higher (*P ≤ *0.02) than that of HRPG. The proportion of propionate in MRPG group was significantly higher than LRPG group. Supplementing RPG with different doses had a tendency (*P* < 0.1) to change rumen pH and the concentration of NH_3_-N. The acetate/propionate ratio was not affected (*P* = 0.62) by the treatments (Table 1).

**TABLE 1:** Rumen fermentation parameters affected by different doses of RPG

Parameter	Treatment^*a*^				SEM	*P*
	CON	LRPG	MRPG	HRPG		
pH	6.13	6.20	6.10	6.34	0.19	0.080
NH_3_-N concn (mg/dl)	11.18	13.93	11.10	11.37	2.29	0.057
TVFA concn (mM)	107.93^AB^	96.41^B^	118.08^A^	98.98^B^	11.69	0.004
						
VFA						
mol% TVFA						
Acetate	69.66^AB^	60.69^B^	75.19^A^	63.51^B^	7.97	0.005
Propionate	25.37^AB^	23.70^AB^	28.59^A^	23.05^B^	3.58	0.021
Butyrate	9.71^AB^	9.02^B^	10.98^A^	9.27^B^	0.98	0.002
Isobutyrate	0.67	0.64	0.70	0.65	0.08	0.435
Valerate	1.20	1.17	1.32	1.20	0.17	0.305
Isovalerate	1.33	1.20	1.31	1.29	0.18	0.510
Acetate:propionate^*b*^	2.75	2.62	2.66	2.78	0.27	0.620

a CON, control group, a basal diet; LRPG, low RPG, a basal diet plus 200 g/day RPG; MRPG, medium RPG, a basal diet plus 350 g/day RPG; HRPG, high RPG, a basal diet plus 500 g/day RPG. Means in a row with different superscript capital letters differ significantly.

b The ratio of acetate mol% TVFA to propionate mol% TVFA.

### Changes of ruminal bacteria.

**FIG 1:**
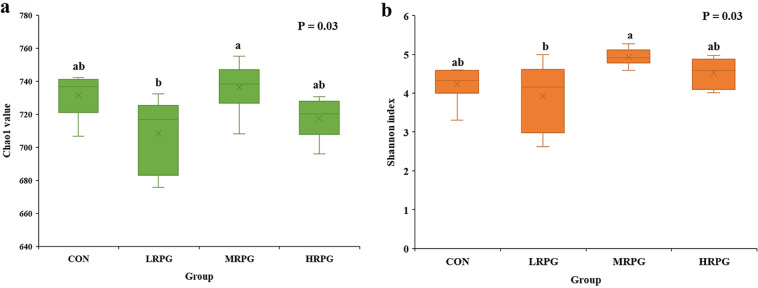
Differences in ruminal bacterial diversity and richness as a result of CON, LRPG, MRPG, and HRPG. (a) Bacterial richness estimated by the Chao1 value. (b) Bacterial diversity estimated by the Shannon index. CON, control group, a basal diet; LRPG, low RPG, a basal diet plus 200 g/day RPG; MRPG, medium RPG, a basal diet plus 350 g/day RPG; HRPG, high RPG, a basal diet plus 500 g/day RPG. Lowercase letters (a and b) above bars indicate significant differences between groups; bars that do not share the same letter are significantly different from each other (*P* < 0.05).

Through the bacterial 16S rRNA sequencing, a total of 1,668,176 raw reads were obtained in the four groups. After screening, 1,472,852 valid reads were obtained, accounting for 88.3% of the raw reads. Sample-based rarefaction curves (see Fig. S1 in the supplemental material) showed that the OTU numbers increased, along with the increase in sample number. As the number of reads per sample increased, the rate of new operational taxonomic unit (OTU) identification diminished, which implies that the sampling depth was sufficient to evaluate dominant members of the rumen bacterial community. The curve became asymptotically stable, along with the saturation of OTU number, indicating that the sequencing depth was adequate to accurately represent rumen bacterial composition. Similarly, the Good’s coverage value of all samples exceeded 99.5%, which demonstrated the accuracy and reproducibility of the sequencing. A Venn diagram showed the shared reads numbers among four groups (see Fig. S2). A total of 752 OTUs were identified in the four groups, among which 746 OTUs were found across all groups and accounted for 99.2% of the total OTUs, indicating the existence of a large common microbiome. OTU1, which was classified into the family of *Succinivibrionaceae*, was the predominant OTU, making up 17.97% of the total OTUs. According to the Chao1 value (*P* = 0.03) and Shannon index (*P* = 0.03), there were significant differences in the richness and diversity of microbial communities between LRPG and MRPG (Fig. 1).

**FIG 2:**
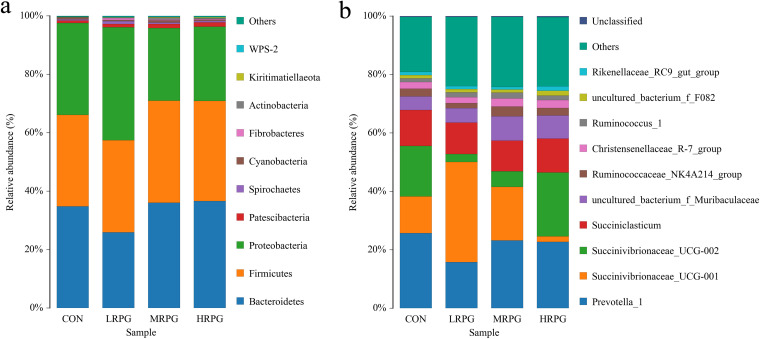
Distribution of the bacterial community composition across the four treatments. (a) Phylum level; (b) genus level. CON, control group, a basal diet; LRPG, low RPG, a basal diet plus 200 g/day RPG; MRPG, medium RPG, a basal diet plus 350 g/day RPG; HRPG, high RPG, a basal diet plus 500 g/day RPG.

**FIG 3:**
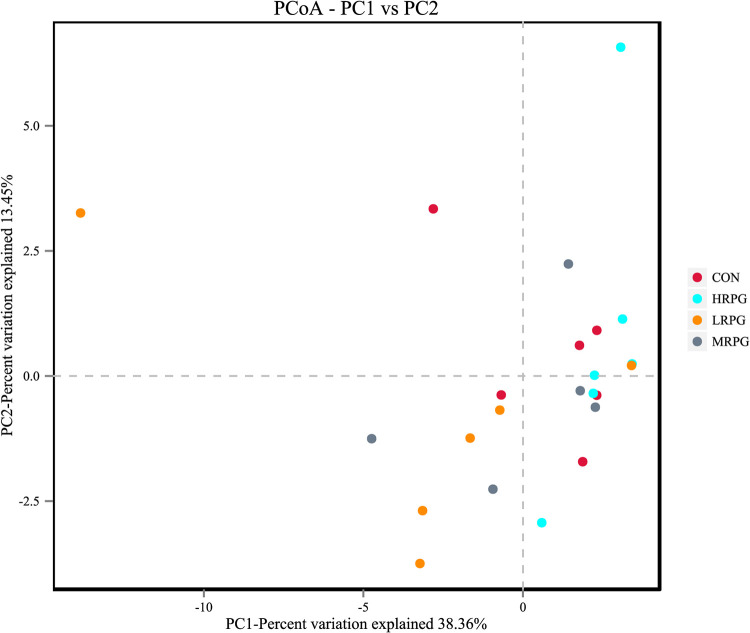
Unweighted principal coordinate analysis (PCoA) of rumen bacteria microbiota. CON, control group, a basal diet; LRPG, low RPG, a basal diet plus 200 g/day RPG; MRPG, medium RPG, a basal diet plus 350 g/day RPG; HRPG, high RPG, a basal diet plus 500 g/day RPG.

There were 16 bacterial phyla identified in the rumen samples. Among these phyla, *Bacteroidetes*, *Firmicutes*, and *Proteobacteria* showed relatively high abundance, with percentages of 32.78% ± 0.21% (mean ± the standard error of the mean), 32.77% ± 0.52%, and 30.97% ± 3.87%, respectively (Fig. 2a). As the level of RPG addition increased, the relative abundance of *Fusobacteria* (*P* < 0.01) decreased (Fig. 3). At the genus level, there were 133 bacterial genera identified through analysis of microbiota compositions, and 94 genera were present in all samples, which represented the core microbiome in this study. Among these genera, *Prevotella* (21.62% ±3.69%), *Succinivibrionaceae_UCG-001* (17.97% ± 2.37%), *Succinivibrionaceae_UCG-002* (11.47% ± 5.05%), *Succiniclasticum* (11.34% ± 0.71%), *uncultured_bacterium_f_Muribaculaceae* (6.17% ± 1.67%), *Ruminococcaceae_NK4A214_group* (2.49% ± 0.56%), *Christensenellaceae_R-7_group* (2.38% ± 0.33%), *Ruminococcus* (1.61% ± 0.28%), *uncultured_bacterium_f_F082* (1.22% ± 0.25%), and *Rikenellaceae_RC9_gut_group* (1.13% ± 0.24%) were considered high-abundance taxa (Fig. 2b).

Among the genera with significantly different abundance between treatments, the RPG supplement groups increased the relative abundance of the bacteria at different degrees. Five genera (*Ruminococcus*, *Lachnospiraceae_NK3A20_group*, *Ruminiclostridium*, *Lachnospiraceae_UCG-008*, and *Olsenella*) were significantly higher (*P* < 0.03) in MRPG compared to CON and LRPG. Five genera (*Pseudobutyrivibrio*, *Ruminococcaceae_UCG-005*, *[Eubacterium]_coprostanoligenes_group*, *Rikenellaceae_RC9_gut_group*, and *Succinivibrionaceae_UCG-002*), three genera (*Oribacterium*, *Succinivibrionaceae_UCG-001*, and *Fibrobacter*), and two genera (*Prevotella* and *Ruminobacter*) had the highest relative abundances (*P* < 0.05, *P ≤ *0.01, and *P* < 0.02, respectively) in HRPG, LRPG, and CON groups, respectively, among all treatments (Table 2).

**TABLE 2:** Main microbiota that significantly changed between the four groups^*a*^

Phylum	Genus	Abundance (%) for various treatments^*b*^				SEM	*P*
		CON	LRPG	MRPG	HRPG		
*Firmicutes*	*Ruminococcus*	1.27^B^	1.72^AB^	1.99^A^	1.58^AB^	0.44	0.021
	*Pseudobutyrivibrio*	0.52^B^	0.70^AB^	0.80^AB^	0.94^A^	0.30	0.068
	*Lachnospiraceae_NK3A20_group*	0.46^B^	0.50^B^	1.08^A^	0.68^AB^	0.38	0.011
	*Ruminococcaceae_UCG-005*	0.43^AB^	0.29^B^	0.36^AB^	0.62^A^	0.21	0.035
	*Oribacterium*	0.22^B^	0.58^A^	0.46^AB^	0.31^B^	0.18	0.003
	*Ruminiclostridium*	0.39^AB^	0.20^B^	0.62^A^	0.29^B^	0.23	0.005
	*Lachnospiraceae_UCG-008*	0.24^B^	0.40^AB^	0.41^A^	0.38^AB^	0.12	0.026
	*[Eubacterium]_coprostanoligenes_group*	0.14^AB^	0.12^B^	0.15^AB^	0.20^A^	0.06	0.059
*Bacteroidetes*	*Prevotella*	25.91^A^	16.09^B^	23.31^AB^	22.67^AB^	6.00	0.019
	*Rikenellaceae_RC9_gut_group*	1.10^AB^	1.05^AB^	0.98^B^	1.55^A^	0.38	0.026
*Proteobacteria*	*Succinivibrionaceae_UCG-001*	11.24^B^	32.76^A^	14.79^AB^	1.60^B^	14.94	0.003
	*Succinivibrionaceae_UCG-002*	17.59^A^	2.99^B^	6.05^B^	22.62^A^	8.30	0.001
	*Ruminobacter*	0.68^A^	0.13^B^	0.16^B^	0.42^AB^	0.33	0.006
*Actinobacteria*	*Olsenella*	0.08^B^	0.08^B^	0.58^A^	0.22^AB^	0.33	0.014
*Fibrobacteres*	*Fibrobacter*	0.09^B^	0.60^A^	0.27^AB^	0.28^AB^	0.29	0.011

a Main microbiota account for ≥0.05% of the total sequences in at least one of the samples. The abundance of each genus is expressed as a percentage.

b Means in a row with different superscript capital letters differ significantly. CON, control group, a basal diet; LRPG, low RPG, a basal diet plus 200 g/day RPG; MRPG, medium RPG, a basal diet plus 350 g/day RPG; HRPG, high RPG, a basal diet plus 500 g/day RPG.

**FIG 4:**
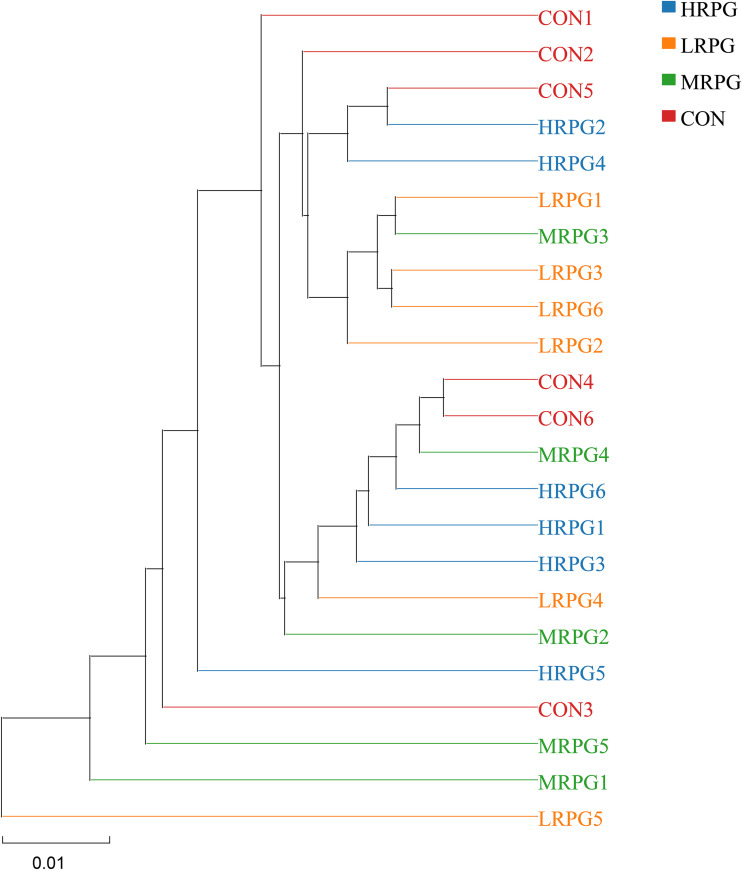
UPGMA clustering tree of rumen bacterial microbiota. The closer the samples are, the shorter the branch length and the more similar the species compositions of the two samples. CON, control group, a basal diet; LRPG, low RPG, a basal diet plus 200 g/day RPG; MRPG, medium RPG, a basal diet plus 350 g/day RPG; HRPG, high RPG, a basal diet plus 500 g/day RPG.

Furthermore, a principal coordinate analysis (PCoA) plot and an unweighted pair-group method with arithmetic mean (UPGMA) clustering tree with the unweighted UniFrac matrix distances were not largely separated from each other at the OTU level, supporting that the RPG supplementation did not significantly influence the bacterial community composition (Fig. 3 and 4).

### Rumen metabolomics profiling.

As shown in Fig. S3, a total of 315 metabolites were identified and quantified in the ruminal fluid from the four groups (see Table S3) according to querying the Human Metabolome Database (HMDB) (http://www.hmdb.ca). The four groups shared the same metabolite categories, including lipids and lipid-like molecules, organic acids and derivatives, organoheterocyclic compounds, and organic oxygen compounds.

The LC-MS spectral data among the four groups, including the QC samples used throughout the analysis, were first analyzed using a principal-component analysis (PCA) score plot (see Fig. S4) to provide a global overview of the differences among the metabolite data. Figure S5a, c, e, g, i, and k shows the orthogonal partial least-squares discriminant analysis (OPLS-DA) score plots, which were determined to verify the differentiated metabolites between groups and supervise the multivariate analysis. All the samples in the score plots were within the 95% Hotelling T2 ellipse, and clear separation and discrimination between groups are evident. The validation plots after permutation test (see Fig. S5b, d, f, h, j, and l) represented the OPLS-DA model assessment parameters in distinguishing the four groups. The corresponding *R*^2^ values for the OPLS-DA models in CON versus LRPG, CON versus MRPG, CON versus HRPG, LRPG versus MRPG, LRPG versus HRPG, and MRPG versus HRPG were 0.951, 0.857, 0.853, 0.945, 0.977, and 0.951, respectively. The permutation tests of the four groups were all in a good range with the *R*^2^ values of the four groups were all >0.853, indicating that the model has satisfactory validity and could be used to identify the difference between the two treatments.

**FIG 5:**
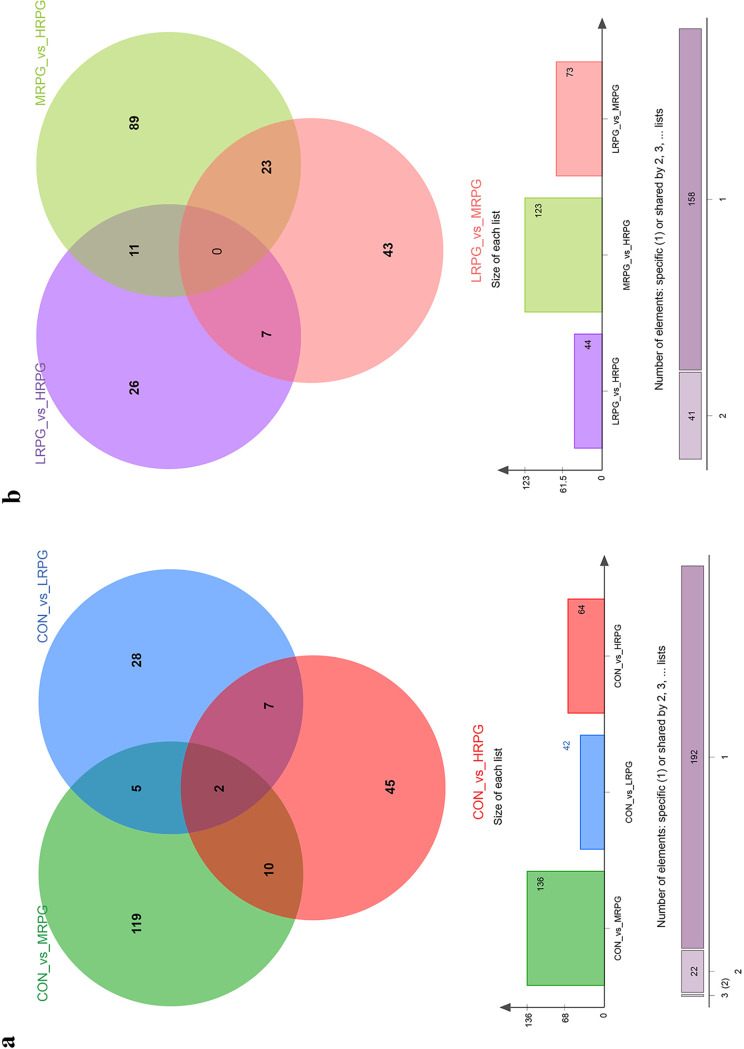
Venn diagrams demonstrate overlap of differential metabolites for CON, LRPG, MRPG, and HRPG group. (a) CON versus LRPG, MRPG, and HRPG; (b) pairwise comparison between LRPG, MRPG, and HRPG. CON, control group, a basal diet; LRPG, low RPG, a basal diet plus 200 g/day RPG; MRPG, medium RPG, a basal diet plus 350 g/day RPG; HRPG, high RPG, a basal diet plus 500 g/day RPG.

**FIG 6:**
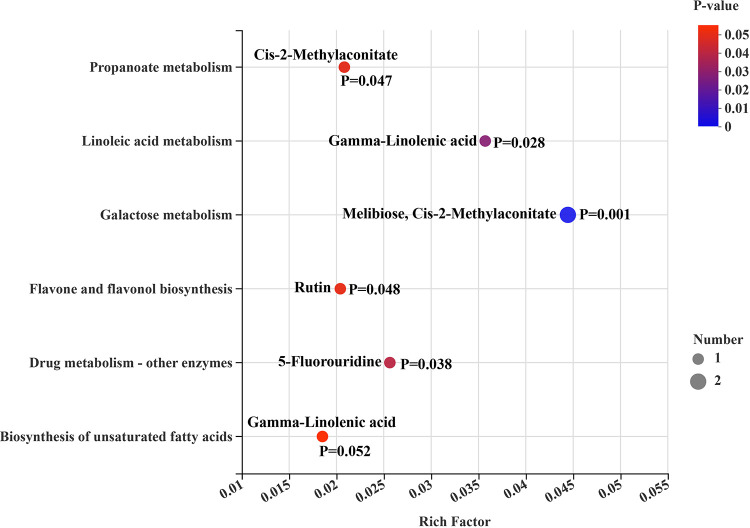
Differential metabolites KEGG pathway enrichment analysis. The *y* axis indicates the name and classification of the pathway; the *x* axis represents the rich factor, the higher the value, the greater the degree of enrichment. Color is used to distinguish the enrichment significance (*P* value). The darker the color, the more significantly the metabolic pathway is enriched. The color gradient on the right represents the value of *P*. Differential metabolites and *P* values are labeled around each dot.

In total, 42 differential metabolites (VIP > 2 and *P* < 0.05) were obtained from the comparisons of CON versus LRPG, CON versus MRPG, CON versus HRPG, LRPG versus MRPG, LRPG versus HRPG, and MRPG versus HRPG (see Table S4). Among them, 18, 5, 4, 3, 3, 1, 1, and 1 metabolite were classified into lipids and lipid-like molecules (the super class level is the same as below), organoheterocyclic compounds, organic acids and derivatives, organic oxygen compounds, phenylpropanoids and polyketides, benzenoids, hydrocarbon derivatives, and nucleosides, nucleotides, and analogues, respectively. Five metabolites were unclassified or listed in Fig. S6 in the supplemental material. Comparison among different treatments found that 18 metabolites had the highest in the MRPG group (*P* < 0.05), among which 13 metabolites [(*R*)-8-acetoxycarvotanacetone, isopersin, 10-hydroxy-(2E,8E)-decadien-4-ynoic acid, isoleucyl-lysine, arginyl-proline, histidinyl-isoleucine, *cis*-2-methylaconitate, taxifolin 3-arabinoside, 5-fluorouridine, (*R*)-11,12,13-Trinor-1(5),6,9-guaiatrien-8-one, *N*-acetyl-dl-tryptophan, prostaglandin D1 alcohol, and β-d-glucopyranosiduronic acid], 4 metabolites [physalolactone B, 5-hydroxyvalproic acid, (*Z*)-3-nonen-1-ol, and *O*-desmethylvenlafaxine glucuronide], and 1 metabolite (naringenin 5-rhamnoside) had the lowest levels in the CON group, the LRPG group, and the HRPG group, respectively (see Table S5). Similarly, a shown in a Venn diagram indicating the specific and common shared differential metabolites in different comparison pairs, MRPG had the highest number of specific metabolites (Fig. 5). The CON_vs_MRPG had the highest number of unique differential metabolites (119 metabolites), followed by the CON_vs_HRPG (45 metabolites) and the CON_vs_LRPG (28 metabolites) (Fig. 5a). In addition, the difference between MRPG and HRPG was more prominent (Fig. 5b). As shown in Fig. 6, differential metabolites were mainly enriched in the following metabolic pathways: galactose metabolism, linoleic acid metabolism, drug metabolism–other enzymes, propanoate metabolism, flavone and flavonol biosynthesis, and biosynthesis of unsaturated fatty acids.

### Correlations between the ruminal microbiome and metabolome.

**FIG 7:**
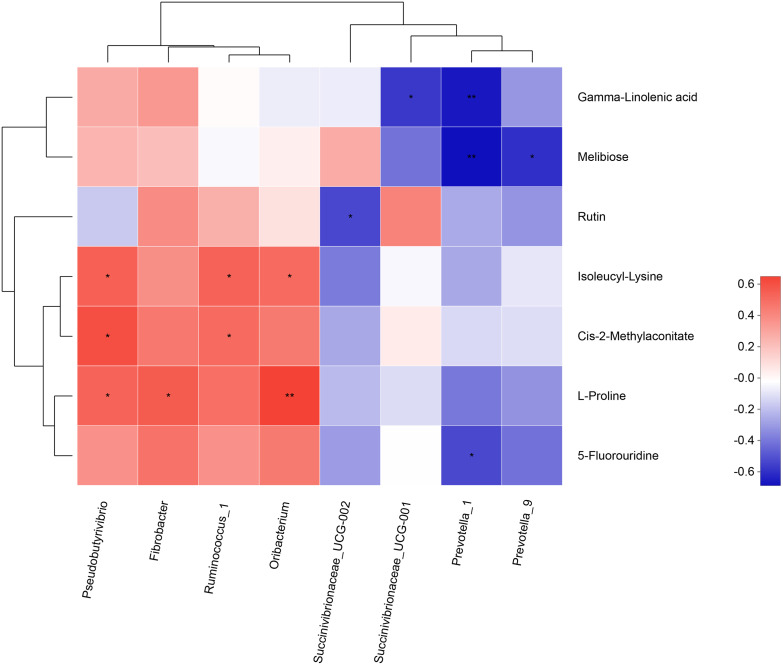
Correlation analysis between microbiota and metabolites affected by the RPG dose. Each column represents a microbe, each row represents a metabolite, and each colored box represents a Spearman correlation coefficient between a microbe and a metabolite. Red represents a positive correlation, and blue represents a negative correlation. *, significant correlation (*P* < 0.05). A dendrogram of metabolite clustering is shown on the left, and a dendrogram of microbe clustering is shown at the top.

Correlation analysis was performed between eight microbial genera and seven differential metabolites that met the screening conditions. As shown in Fig. 7, *Pseudobutyrivibrio* was positively associated (*r* > 0.53, *P* < 0.03) with isoleucyl-lysine, *cis*-2-methylaconitate, and l-proline. *Fibrobacter* was positively associated with l-proline (*r* = 0.56, *P* = 0.02). *Ruminococcus* was positively associated (*r* > 0.51, *P* < 0.04) with isoleucyl-lysine and *cis*-2-methylaconitate. *Oribacterium* was positively associated (*r* > 0.51, *P* < 0.02) with isoleucyl-lysine and l-proline. *Succinivibrionaceae_UCG-002* was negatively associated with rutin (*r* = −0.53, *P* = 0.04). *Succinivibrionaceae_UCG-001* was negatively associated with gamma-linolenic acid (*r* = −0.57, *P* = 0.02). *Prevotella* was negatively associated (*r* < −0.53, *P* < 0.04) with gamma-linolenic acid, melibiose, and 5-fluorouridine. *Prevotella* was negatively associated with melibiose (*r* = −0.60, *P* = 0.01).

## DISCUSSION

This study characterized the changes of rumen microbial flora and evaluated the shift in rumen metabolism in response to extra supplementation of RPG by 16S gene sequencing and LC-MS methods, respectively, and further uncovered the relationship between them through correlation analysis, which was helpful for understanding the effects of RPG utilization on dairy cows.

The phylum-level core microbiomes were *Bacteroidetes*, *Firmicutes*, and *Proteobacteria*, and the genus-level dominant bacteria were *Prevotella*, *Succinivibrionaceae*, *Succiniclasticum*, *Ruminococcaceae_NK4A214_group*, *Christensenellaceae_R-7_group*, *Ruminococcus*, *Rikenellaceae_RC9_gut_group*, etc., which is consistent with the findings of previous studies (7, 16, 21). Among these organisms, *Firmicutes* plays an important role in the degradation of fiber and cellulose, whereas the main function of *Bacteroidetes* is to degrade carbohydrates and proteins and to promote the development of the gastrointestinal immune system (22). Two major phyla, *Firmicutes* and *Bacteroidetes*, have been shown to be involved in lipid and bile acid metabolism, and the abatement of the *Firmicutes*/*Bacteroidetes* ratio is closely related to fat deposition (23), which helps to maintain systemic energy homeostasis in host as gut microbiota (24). Intriguingly, removal of *Firmicutes* and *Bacteroidetes* increased glucagon-like peptide-1 (GLP-1) secretion in parallel with alterations in levels of beneficial gut metabolites, which appear to mediate the improvement in insulin sensitivity (25). The diversity and richness of the bacterial community can be influenced by feed (16, 17). In the present study, the MRPG group showed significantly improved diversity and richness of rumen microbiota. Thus, RPG supplementation could significantly change the alpha diversity of rumen microbiota, and the changes in the *Firmicutes*/*Bacteroidetes* ratio among groups show that moderate RPG supplementation could allow dairy cows to reasonably allocate the energy obtained to maintenance and production needs, avoiding excessive energy consumption, which would aggravate weight loss, especially MRPG.

*Prevotella*, *Ruminococcus*, *Eubacterium*, and other members of the *Lachnospiraceae* and *Ruminococcaceae* families are widely recognized as members of the core rumen microbiota (≥90% of samples) (17, 26). In the gastrointestinal environment, members of the *Lachnospiraceae* and *Ruminococcaceae* were able to degrade plant cellulose and hemicellulose that are indigestible by the host and convert them into short-chain fatty acids (SCFA; mainly acetate, butyrate, and propionate) that can be absorbed and used for energy by the host (21, 27). Many members of *Lachnospiraceae* were characterized by their cellulose-decomposing activity (28). As the most abundant family in the *Firmicutes*, the *Ruminococcaceae* have moderate heritability. This family is composed of both fibrolytic organisms and members involved in starch hydrolysis, which could produce acetate, formate, succinate, and so on (29). In the present study, compared to other groups, *Lachnospiraceae_NK3A20_group*, *Lachnospiraceae_UCG-008*, *Ruminiclostridium*, and *Ruminococcus* showed the highest abundance in the MRPG group, which could partly explain the highest concentrations of SCFA such as acetate, butyrate, and propionate in rumen fluid in the MRPG group. Furthermore, *Lachnospiraceae* and *Ruminococcaceae* were two of the most abundant families from the order *Clostridiales* found in the mammalian gastrointestinal environment and involved in maintaining gastrointestinal health (30). The family *Ruminococcaceae* to which *Ruminiclostridium* belongs is regarded as potentially beneficial bacteria because it participated in the positive regulation of the intestinal environment related to the release of inflammatory and cytotoxic factors from the intestine and linked to immune regulation and healthy homeostasis (31). These findings suggest that MRPG significantly increased the abundance of *Lachnospiraceae* and *Ruminococcaceae*, leading to increased levels of SCFA in the rumen. MRPG supplementation may also inhibit the release of inflammatory and cytotoxic factors and maintain the stability of the rumen internal environment by increasing the abundance of *Lachnospiraceae* and *Ruminococcaceae*.

*Prevotella* is a known starch and protein utilization bacterium (32), has the highest abundance in the rumen of lactating dairy cows, and may be altered in responding to different diets and feeding patterns (33, 34). *Prevotella* has enzymes that degrade xylan and other hemicelluloses into SCFA, such as acetate, propionate, or propionate-precursor succinate (28). Here, the *Prevotella* in the LRPG group was at the lowest level and was consistent with the lower VFA in LRPG. The *Rikenellaceae_RC9_gut_group* and *Ruminococcaceae_UCG-005* could be used as signals for conventional diarrhea, since their abundances were obviously decreased after diarrhea (35). *Rikenellaceae* were correlated with resistance to the development of colitis after CTLA-4 (cytotoxic T lymphocyte-associated antigen 4) blockade and could limit inflammation by stimulating T-regulatory cell differentiation (36). When the immune system or intestinal microbiota is dysfunctional, these two bacteria may cause endogenous infection (35). In the present study, both *Rikenellaceae_RC9_gut_group* and *Ruminococcaceae_UCG-005* in the HRPG group were at the highest level, indicating that HRPG supplementation may help regulate lymphocyte differentiation and the release of anti-inflammatory factors by regulating microorganisms, so as to maintain homeostasis and prevent diarrhea. However, how the rumen microbiota directly or indirectly regulates diarrhea through intestinal microbiota remains to be further studied. Based on these findings, LRPG supplementation reduced the abundance of *Prevotella* and the concentrations of SCFA, indicating that the degradation of starch was hindered. HRPG supplementation could improve the abundance of inflammation-related bacteria and may play a role in the balance of rumen microecology.

The metabolites of ruminal fluid may be the intermediates of the exchange between the rumen microflora and the host. Li et al. (37) revealed that some ruminal fluid metabolites were associated with different levels of microbiota. The main differential metabolites here were fatty acids and conjugates, amino acids, peptides, and analogues, which is consistent with the Li et al. study (37). Microbes in the gastrointestinal tract, particularly in the rumen, can produce SCFA such as acetate, propionate, and butyrate, from undigested dietary polysaccharides in cattle, which could contribute up to 70% of the energy intake (28) and stimulate the growth of the rumen epithelium (38). Therefore, we found that RPG supplementation significantly affected rumen fermentation indexes such as VFA and NH_3_-N and may promote rumen microbial lipid metabolism and amino acid metabolism. However, more studies are needed to investigate the specific influencing mechanism of RPG on ruminal microbiota in combination with metagenomics, lipidomics, and proteomics.

Isoleucyl-lysine, arginyl-proline, and histidinyl-isoleucine are all dipeptides. They are incomplete breakdown products of protein digestion or protein catabolism. Peptide carbon is more efficiently converted into bacterial protein than amino acid carbon. Cotta et al. (39) conducted mixed culture experiments of free amino acids and peptides *in vitro* and found that microorganisms were more likely to ingest peptides than amino acids. Most rumen peptides are catabolic, releasing dipeptides or tripeptides rather than free amino acids from the N terminus of the polypeptide through the activity of dipeptidyl peptidase secreted by *Bacteroidetes* in the rumen. Rumen microorganisms utilize tryptic peptides from *Chlorella* protein, forming carbon dioxide, volatile fatty acids, and bacterial proteins (40). *cis*-2-Methylaconitate belongs to carboxylic acids and derivatives, which are involved in the carbohydrate metabolism of the organism and are mainly related to enzymes in propionate catabolism via the methylcitrate pathway. The 2-methylcitric acid cycle is a common route of propionate catabolism in microorganisms and participates in the tricarboxylic acid cycle to provide energy (41). The concentrations of the four organic acid mentioned above were significantly changed by treatments showing the highest levels in the MRPG group, indicating that MRPG provided more effective microbial protein precursors and promoted propionate catabolism pathway. Bacteria in the phylum *Firmicutes*, such as *Ruminococcus* and *Pseudobutyrivibrio*, were negatively correlated with isoleucyl-lysine, *cis*-2-methylaconitate, and l-proline to various degrees. At the genus level, *Ruminococcus* was considered to be a member of the core gastrointestinal tract microbiota (≥90% of samples) of cattle (17, 42). Members of the *Ruminococcus* genus found in the bovine rumen are cellulolytic and produce acetate, formate, and succinate from cellulose (28). This can be partly explained because *Ruminococcus* was found to be positively associated with *cis*-2-methylaconitate in the present study. *Pseudobutyrivibrio* was mainly involved in the metabolism of fiber and other carbohydrates and could decompose feed to produce carboxylic acids and derivatives, which helps to explain why *Pseudobutyrivibrio* was positively related to *cis*-2-methylaconitate in the present study. Among amino acids, proline is known to play an important role in conceptus growth and in development and gastrointestinal tract microbiota reequilibration in the case of dysbiosis (43). As a major amino acid for maintaining the function and structure of cells, l-proline is also an important regulator of cell metabolism and physiology. Moreover, l-proline also plays an important role in protein synthesis and structure, nutrition, and metabolism and in antioxidant and immune responses in wounds (44). Correlation analysis results showed that *Pseudobutyrivibrio*, *Fibrobacter*, and *Oribacterium* are significantly positively correlated with l-proline. Thus, MRPG supplementation could affect organic acid metabolism in the rumen and positively regulate energy metabolism pathways through carbohydrate-related decomposers. Meanwhile, it may promote gastrointestinal microbiota homeostasis and contribute to the synthesis of bacterial proteins.

NH_3_-N in the rumen was one of the products of rumen fermentation and was also the raw material for the synthesis of microbial proteins by rumen microbes, reflecting the balance between the production of NH_3_-N by the decomposition of crude protein in feed by rumen microbes and the synthesis of microbial protein using NH_3_-N. Rutin has the effect of reducing the efficiency of microbial protein synthesis (45), so the concentration of rutin has a positive correlation with the concentration of NH_3_-N in rumen fluid. Melibiose is a dicarbohydrate composed of galactose and glucose. Kyoto Encyclopedia of Genes and Genomes (KEGG) pathway analysis showed that melibiose, which involved in carbohydrate metabolism such as galactose metabolism and flavone and flavonol biosynthesis, was negatively correlated with *Prevotella*, suggesting that *Prevotella* was involved in the melibiose catabolism. Gamma-linolenic acid is mainly involved in lipid metabolism, and linoleic acid and linolenic acid are common unsaturated essential fatty acids. A previous study (46) confirmed that unsaturated fatty acids were toxic to ruminal bacteria. *Pseudobutyrivibrio*, a butyrate-producing bacterium from the rumen, seemed to resemble Butyrivibrio fibrisolvens closely in phenotype. In this study there was no significant correlation between gamma-linoleic acid and *Pseudobutyrivibrio* in the HRPG group, which is consistent with the study of Maia et al. (46), which revealed the toxicity of unsaturated fatty acids to the biohydrogenating ruminal bacterium B. fibrisolvens. Biohydrogenation is a detoxification mechanism and can enable *Butyrivibrio* to survive the bacteriostatic effects of polyunsaturated fatty acids (46). KEGG pathway enrichment analysis results showed that RPG supplementation promoted linoleic acid metabolism and biosynthesis of unsaturated fatty acids (UFA), suggesting that RPG contributes to upregulate the UFA synthesis pathway, thus promoting the growth of beneficial bacteria. In the present study, the genus *Prevotella* was also significantly negatively correlated with gamma-linolenic acid, suggesting that *Prevotella* may be related to the hydrogenation of unsaturated fatty acids, which is in agreement with work by Huws et al. (47), who reported that as-yet-uncultured bacteria classified as *Prevotella*, *Lachnospiraceae* incertae sedis, and unclassified *Clostridiales* and *Ruminococcaceae* may play a role in biohydrogenation. Taken together, these findings show that HRPG facilitated the hydrogenation of microorganisms and also lipid metabolism. LRPG supplementation could promote rumen microbial activity and rumen epithelial cell function and affect protein metabolism in the rumen by increasing the concentration of rutin.

Compared to both control and other RPG groups, MRPG thus significantly increased the abundance of beneficial bacteria, reduced lipolysis, and improved amino acid metabolism. These findings are in line with our previous study, in which the results of lactation performance, serum biochemical indexes, and serum metabolomics analysis showed that MRPG significantly improved the indexes related to NEB, such as NEFA, BHBA, AST and GLU, and alleviated the lipolysis effect (48). This was not dose dependent on RPG supplementation and was associated with the high-glucose and high-fat side effects of high-dose RPG supplementation. Vanhatalo et al. pointed out that the positive responses to incremental glucose supply may be related to a quantitatively increased glucose need, especially at the early stage of lactation. Moreover, when the glucose supply exceeds a certain amount, it has a negative effect. Further studies are needed to quantify lactational and metabolic responses as a function of graded amounts of added glucose on diets (49). Similarly, with the increase in dietary fat, the mean total volatile fatty acids in the rumen and the propionate concentration decreased in a linear fashion (50), and the protozoan numbers also demonstrated a linear and quadratic negative correlation (51). The reduction was most severe at the highest level of fat supplementation (52).

In conclusion, we examined here the effects of different doses of RPG supplementation on rumen microbes and metabolism. Compared to other groups, MRPG supplementation has outstanding advantages, significantly increasing the richness and diversity of rumen bacteria, improving the abundance of beneficial bacteria, and promoting fat metabolism and organic acid production in the rumen, and thus promotes rumen system development, microbial growth, and energy metabolism. Through the analysis of rumen microbes, metabolites, and their interactions, we have demonstrated the role of RPG supplement on rumen function of dairy cows and provided a basis for the widespread application of RPG in early-lactation dairy cows. However, the overall impact of RPG on the digestion and metabolism of cows needs to be analyzed in combination with hindgut microbes and metabolism, which will be the focus of our future research.

## MATERIALS AND METHODS

### Ethics approval and consent to participate.

All experimental designs and protocols involving animals were approved by the Animal Ethics Committee of the Chinese Academy of Agricultural Sciences, Beijing, People’s Republic of China (approval IAS2019-54) and complied with the recommendations of the academy’s animal research guidelines.

### Animals, diets, and experimental design.

Twenty-four multiparous Holstein cows in early lactation, blocked by parity (2, *n* = 12; 3, *n* = 8; 4, *n* = 4; evenly distributed to four groups) and milk yield (35.78 ± 7.90 kg/day), were randomly assigned to control (CON), low-RPG (LRPG), medium-RPG (MRPG) or high-RPG (HRPG) groups in a randomized block design. The cows were fed a basal total mixed ration diet supplemented with 0, 200, 350, and 500 g of RPG per cow per day, respectively, from calving to 35 days postpartum with the first 7 days for adaptation. The RPG dosage was based on the dosage requirements provided by the manufacturer (200 to 500 g/cow/day) and a previous study, in which Li et al. (4) indicated that 200-g dietary RPG supplementation seemed to be insufficient for the elevated energy consumption combined with increased milk production. The RPG used in this study adopted the latest international rumen enveloping technology, and the rumen protection level of the product was 54.03% (6). It was prepared with 45% glucose as the core material, 45% hydrogenated fat by mass as the coating, and 10% water (4). Therefore, cows from the CON, LRPG, MRPG, or HRPG group would receive 0, 90, 157.5, or 225 g of glucose supplement per day, respectively.

The experiment was conducted in Beijing Dairy Cattle Center (Beijing, China). The RPG was equally distributed three times a day and top dressed on the total mixed rations, which were formulated based on the National Research Council guidelines (NRC, 2001). The ingredients and chemical composition of the diets during the experiment are shown in Table S1 in the supplemental material. During the experiment periods, cows were fed three times daily at 07:00, 14:00, and 21:00 and milked three times daily at 06:00, 13:00, and 20:00, respectively. Cows were housed in individual stalls, and fresh water was provided *ad libitum*.

### Ruminal fluid sample collection and measurements.

At the end of the experiment (day 35), ruminal fluid samples were collected using an oral stomach tube connected to a vacuum pump through the esophagus before the afternoon feeding, as described by Shen et al. (53). Somce of the collected samples were immediately frozen in liquid nitrogen and stored at −80°C for further DNA extraction, and some of the samples used for metabolomic analysis were filtered through four layers of cheesecloth first and then subjected to the same procedures; unfortunately, a rumen fluid sample used to determine the 16S rRNA portion was damaged. The rest of the collected samples were immediately measured with a portable pH meter (Testo-205; Testo AG, Lenzkirch, Germany) and filtered by using four layers of cheesecloth, and then meta-phosphoric acid (10 ml of filtrate added with 1 ml of 250-g/liter meta-phosphoric acid) was added for the determination of volatile fatty acid (VFA) and ammonia nitrogen (NH_3_-N) concentrations. The VFA profile was determined with a gas chromatograph (Agilent Technologies, Inc., Wilmington, DE) (54), and the NH_3_-N concentration was analyzed using a phenol-hypochlorite assay by visible spectrophotometry (Secomam, Domont, France) (55).

A randomized block design with repeated measures was used. The rumen fermentation data for the cows were analyzed with PROC MIXED (SAS 9.2; SAS Institute, Inc., Cary, NC). Duncan’ s multiple-range test was used for this experiment. The results are presented as least-square means. The statistical significance was determined at *P ≤ *0.05, and trends were declared at 0.05 < *P ≤ *0.10.

### DNA extraction, 16S rRNA gene amplification, and sequencing.

Microbial genomic DNA was extracted from ruminal fluid samples using an E.Z.N.A. soil DNA kit (Omega Bio-Tek, Norcross, GA) according to the manufacturer’ s instructions. The DNA extract was checked on a 1% agarose gel, and the DNA concentration and purity were determined with a NanoDrop 2000 UV-vis spectrophotometer (Thermo Scientific, Wilmington, DE). The hypervariable region V3-V4 of the bacterial 16S rRNA gene was amplified using the primer pair 338F (5′-ACTCCTACGGGAGGCAGCAG-3′) and 806R (5′-GGACTACHVGGGTWTCTAAT-3′) by an ABI GeneAmp 9700 PCR thermocycler (ABI, Foster City, CA) (16, 56). The PCR amplification of the 16S rRNA gene was performed as follows: initial denaturation at 95°C for 3 min, followed by 27 cycles of denaturing at 95°C for 30 s, annealing at 55°C for 30 s, and extension at 72°C for 45 s; a single extension at 72°C for 10 min; and finally at 4°C. The PCR mixtures contain 5× TransStart FastPfu buffer (4 μl), 2.5 mM deoxynucleoside triphosphates (2 μl), forward primer (5 μM) at 0.8 μl, reverse primer (5 μM) at 0.8 μl, TransStart FastPfu DNA polymerase (0.4 μl), template DNA (10 ng), and finally ddH_2_O at up to 20 μl. PCRs were performed in triplicate. The PCR product was extracted from a 2% agarose gel and purified using an AxyPrep DNA gel extraction kit (Axygen Biosciences, Union City, CA) according to the manufacturer’s instructions and quantified using a Quantus fluorometer (Promega).

Purified amplicons were pooled as equimolar and pair-end sequenced (2 × 300) on an Illumina MiSeq platform (Illumina, San Diego, CA) according to the standard protocols by Majorbio Bio-Pharm Technology Co., Ltd. (Shanghai, China).

### Sequence processing and analysis.

The raw 16S rRNA gene sequencing reads were demultiplexed and quality filtered by Trimmomatic and merged by FLASH according to the following criteria: (i) 300-bp reads were truncated at any site receiving an average quality score of <20 over a 50-bp sliding window, truncated reads shorter than 50 bp were discarded, and reads containing ambiguous characters were also discarded, and (ii) only overlapping sequences longer than 10 bp were assembled according to their overlapped sequence. The maximum mismatch ratio of overlap region is 0.2. Reads that could not be assembled were discarded. (iii) Each sample’ s sequences were separated based on barcodes (exactly matching) and primers (allowing two nucleotide mismatches), and reads containing ambiguous bases were removed.

OTUs with a 97% similarity cutoff were clustered using UPARSE (v7.1), and chimeric sequences were identified and removed. The taxonomy of each OTU representative sequence was analyzed using RDP Classifier (v2.2; http://rdp.cme.msu.edu/) according to the 16S rRNA database (e.g., Silva SSU128) (57). Alpha-diversity indices of samples were evaluated using mothur (v1.30) (58). The sample rarefaction curve and rank abundance curves were also generated using mothur (v1.30). Beta diversity was estimated by computing the unweighted UniFrac distance and visualized using PCoA and a UPGMA clustering tree, and the results were plotted in QIIME and R (v3.2.4) (59).

### Metabolomics processing.

The ruminal fluid samples were left at room temperature for 1 h to delaminate and then centrifuged at 3,000 × *g* for 10 min at 4°C. Before analysis, the supernatant was added with 400 μl of ice-cold methanol-water (4:1, vol/vol) and then ground with a high-throughput tissue grinder at a low temperature. After vortexing, the samples were sonicated on ice for 10 min three times and then treated at –20°C for 30 min. Subsequently, after a 15-min centrifugation at 13,000 × *g* and 4°C, the supernatants of all samples were collected and transferred to injection vials for further LC-MS analysis.

Ultraperformance liquid chromatography (UPLC) coupled with a triple time-of-flight (TOF) system (AB Sciex, Framingham, MA) was used to analyze the rumen metabolites. The chromatographic separation was performed at 40°C with a BEH C_18_ column (100 mm × 2.1 mm, 1.7 μm; Waters, Milford, CT). The mobile phase consisted of 0.1% formic acid aqueous solution (solvent A) and acetonitrile-isopropanol (vol/vol, 1:1) solution with 0.1% formic acid (solvent B). The injection volume was 20 μl, and the elution gradient is shown in Table S2 in the supplemental material.

MS and MS/MS were performed on a triple TOF mass spectrometer with an electrospray ionization source operated both in positive- and negative-ion modes. The capillary voltage was 1.0 kV, the injection voltage was 40 V, and the collision energy was 6 eV. The source and desolvation temperatures were 120 and 500°C, respectively. The gas flow rate was 900 liters/h. Mass data were collected between 50 and 1,000 *m/z*, and the instrument resolution was 30,000.

### Metabolomics data analysis.

A series of preprocessing of the raw data were required before the statistical analysis. Raw data were imported into Progenesis QI (Waters) for baseline filtering, peak identification and integration, retention time correction, and peak alignment. Thus, the data matrix, including the retention time, *m/z*, and peak intensity, was obtained. The data were then filtered. MS and MS/MS information were matched to the metabolic database. The results are presented in the form of a data matrix.

Metabolomics data files were subjected to multivariate statistical analysis using SIMCA-P 14.0 software (Umetrics, Umeå, Sweden). Data in the data matrix were imported into the software package. Before performing multivariate analysis, data were log transformed and paretoscaled. The overview of global metabolic profiles of all the samples, as revealed by the score plot with an unsupervised multivariate statistical PCA model, was used to reveal similarities and dissimilarities among the intergroups. An OPLS-DA approach was then used to identify the overall differences in metabolic profiles between groups and to find metabolites with different abundance. The OPLS-DA model focused on the desired biological information by filtering out irrelevant information from the discrimination model. In this way, OPLS-DA could better differentiate intergroups and improve the validity of the model. In the OPLS-DA analysis, a “variable importance in projection” (VIP) score that is >1 is the typical rule for selecting differential variables. The permutation test was a random sorting method used to evaluate the accuracy of OPLS-DA models. In order to prevent the model from overfitting, 200 permutation tests were used to examine the fitting effect of the model. A *t* test combined with OPLS-DA multivariate analysis were used to screen for differentially abundant metabolites between groups. The screening criteria were as follows: VIP > 2 and *P* < 0.05. Differential metabolites will be annotated through the KEGG database. KOBAS 2.0 (http://kobas.cbi.pku.edu.cn/) was used to identify statistically significantly enriched pathways according to the Fisher exact test.

### Correlations between microbial communities and rumen metabolites.

Correlations between different metabolites with VIP > 2, *P* < 0.05, a fold change of >2 or <0.5, and differentially abundant microbial genera (*P* < 0.05 and relative abundance > 0.05% in at least one of the samples) were assessed by Spearman’s correlation analysis in R (v3.2.4). Spearman rank correlations were calculated using the Psych packages (FDR correct was embedded in the package) in R. Only connections with a *P* value below 0.05 and |*r*| > 0.5 were considered statistically significant. These correlations were visualized using the pheatmap package in R and Cytoscape (v2.8.2).

### Data availability.

The raw reads supporting the conclusions of this article were deposited in the National Center for Biotechnology Information (NCBI) Sequence Read Archive (SRA) database under study accession number PRJNA645785.

## Supplementary Material

Supplemental file 1

Supplemental file 2

Supplemental file 3
