# Supplementary material for: Ruminal Degradation of Rumen-Protected Glucose Influences the Ruminal Microbiota and Metabolites in Early-Lactation Dairy Cows
Source: Appl Environ Microbiol. 2021 Jan 4;87(2):e01908-20. doi: 10.1128/AEM.01908-20 (PMC7783353; doi:10.1128/AEM.01908-20)

Ruminal degradation of rumen-protected glucose influences ruminal microbiota and metabolites in early lactation dairy cows

Yapin Wang[1], Xuemei Nan[1], Yiguang Zhao[1], Yue Wang[1], Linshu Jiang[2], Benhai Xiong[1,*]

[1] State Key Laboratory of Animal Nutrition, Institute of Animal Science, Chinese Academy of Agricultural Sciences, Beijing 100193, China

[2] Beijing Key Laboratory for Dairy Cow Nutrition, Beijing University of Agriculture, Beijing 102206, China

* Corresponding author. EM: xiongbenhai@caas.cn; Tel: +86-010-62811680; Fax: 010-62815988

Supplementary Material Legends

Supplementary materials include 6 figures and 5 tables.

Fig. S1 Multy samples Rarefaction Curves.

Fig. S2 Venn diagram demonstrates overlap of bacterial OTUs at 97% identity level for CON, LRPG, MRPG, HRPG group. CON, control group, a basal diet; LRPG, low RPG, a basal diet plus 200 g/d RPG; MRPG, medium RPG, a basal diet plus 350 g/d RPG; HRPG, high RPG, a basal diet plus 500 g/d RPG.

Fig. S3 The HMDB Compound Classification of 315 identified metabolites. The names of the HMDB Class level and the percentage of metabolites in order of the number of metabolites were shown. The different colors represented different HMDB classifications, and the area represented the relative proportion of metabolites in this classification.

Fig. S4 Total PCA plot of the ruminal fluid samples corresponding to different diets.

Fig. S5 OPLS-DA score plots and corresponding validation plots derived from the LC-MS metabolite profiles of rumen samples for cows fed increasing doses of RPG supplementary in their diets. OPLS-DA score plots and corresponding validation plots (respectively) for: a, b CON vs. LRPG; c, d CON vs. MRPG; e, f CON vs. HRPG; g, f LRPG vs. MRPG; i, j LRPG vs. HRPG; k, l MRPG vs. HRPG. CON, control group, a basal diet; LRPG, low RPG, a basal diet plus 200 g/d RPG; MRPG, medium RPG, a basal diet plus 350 g/d RPG; HRPG, high RPG, a basal diet plus 500 g/d RPG.

Fig. S6 The HMDB Compound Classification of 80 identified differential metabolites. The names of the HMDB Class level and the percentage of metabolites in order of the number of metabolites were shown. The different colors represented different HMDB classifications, and the area represented the relative proportion of metabolites in this classification.

Table S1 Ingredient and chemical composition of the diets fed to early lactation dairy cows

Table S2 UPLC elution gradient

Table S3 315 Metabolites information overview table

Table S4 42 differential metabolites obtained from the comparisons of CON vs. LRPG, CON vs. MRPG, CON vs. HRPG, LRPG vs. MRPG, LRPG vs. HRPG, and MRPG vs. HRPG. CON, control group, a basal diet; LRPG, low RPG, a basal diet plus 200 g/d RPG; MRPG, medium RPG, a basal diet plus 350 g/d RPG; HRPG, high RPG, a basal diet plus 500 g/d RPG.

Table S5 Metabolites with significant differences between groups

As **Table S3** and **Table S4** are relatively large, we upload them in the form of Table.

Table S1 Ingredient and chemical composition of the diets fed to early lactation dairy cows

**Items**	**Content (g/kg DM)**
Ingredient	
Corn silage	245
Corn ground	157
Alfalfa hay	143
Oat hay	102
Steam-flaked corn	81.7
DDGS	31.1
Soybean meal	123
Rapeseed meal	48.1
Cottonseed meal	33.1
Calcium carbonate	16.3
Salt	5.6
Dicalcium phosphate	9.2
Vitamin and mineral premix	4
Chemical composition	
Crude protein	173
Ether extract	45.6
Neutral detergent fiber	313
Acid detergent fiber	185
Ca	8.9
P	5.2
NEL,MJ/kg	7.13

Table S2 UPLC elution gradient

**Time (min)**	**Flow rate (mL/min)**	**A (%)**	**B (%)**
0	0.4	95	5
3	0.4	80	20
9	0.4	5	95
13	0.4	5	95
13.1	0.4	95	5
16	0.4	95	5

Table S3 315 Metabolites information overview table

Table S4 42 differential metabolites obtained from the comparisons of CON vs. LRPG, CON vs. MRPG, CON vs. HRPG, LRPG vs. MRPG, LRPG vs. HRPG, and MRPG vs. HRPG. CON, control group, a basal diet; LRPG, low RPG, a basal diet plus 200 g/d RPG; MRPG, medium RPG, a basal diet plus 350 g/d RPG; HRPG, high RPG, a basal diet plus 500 g/d RPG.

As **Table S3** and **Table S4** are relatively large, we upload them in the form of Table.

Table S5 Metabolites with significant differences between groups

	ID	Metabolite	Treatments					
			CON	LRPG	MRPG	HRPG	SEM	*P*
Lipids and lipid-like molecules	neg_5046	3-keto-Digoxigenin	1.69	1.15	1.44	1.58	0.17	0.000
	neg_6096	6-Epi-7-isocucurbic acid glucoside	2.60	2.00	2.39	2.50	0.31	0.015
	pos_4049	(3beta,17alpha,23S)-17,23-Epoxy-3,28,29-trihydroxy-27-norlanost-8-en-24-one	2.47	1.89	2.29	2.31	0.33	0.038
	neg_4399	3-Oxohexadecanoic acid	1.90	1.19	1.13	1.49	0.29	0.001
	pos_720	LysoPE(0:0/14:0)	1.79	1.97	1.79	1.37	0.37	0.060
	pos_2595	LysoPE(14:1(9Z)/0:0)	2.59	2.99	2.64	2.35	0.26	0.003
	neg_3130	Physalolactone B	1.47	0.98	1.64	1.54	0.32	0.010
	neg_5665	5-Hydroxyvalproic acid	1.78	1.56	1.95	1.82	0.23	0.048
	pos_4525	Gamma-Linolenic acid	1.45	1.74	1.64	1.75	0.17	0.027
	pos_4514	7(14)-Bisabolene-2,3,10,11-tetrol	1.18	1.72	1.43	1.34	0.31	0.044
	neg_3124	(3beta,5alpha,6alpha,7alpha,22E,24R)-5,6-Epoxyergosta-8,14,22-triene-3,7-diol	1.29	1.75	1.50	1.48	0.19	0.005
	pos_3090	PS(14:0/24:1(15Z))	1.60	2.34	2.13	1.57	0.45	0.017
	pos_4178	Soyasapogenol E	1.16	1.81	1.69	1.14	0.39	0.011
	neg_1660	(R)-8-Acetoxycarvotanacetone	0.78	1.30	1.54	1.06	0.42	0.032
	pos_5110	(Z)-3-Nonen-1-ol	0.83	0.80	1.41	1.17	0.35	0.017

	neg_3081	Isopersin	1.14	1.21	1.67	1.38	0.24	0.005
	pos_2104	10-hydroxy-(2E,8E)-decadien-4-ynoic Acid	2.08	2.17	2.53	2.12	0.15	0.000
	pos_4520	24,25,26,27-Tetranor-23-oxo-hydroxyvitamin D3	1.37	1.88	1.19	1.67	0.28	0.002
Organic acids and derivatives	pos_1798	Isoleucyl-Lysine	0.48	1.24	1.49	0.82	0.42	0.002
	pos_1753	Arginyl-Proline	1.17	1.86	2.21	1.41	0.59	0.029
	pos_5933	Histidinyl-Isoleucine	0.93	1.50	1.79	1.24	0.51	0.046
	neg_1867	Cis-2-Methylaconitate	0.66	1.09	1.26	0.89	0.30	0.015
Organoheterocyclic compounds	neg_2221	1-Phenyl-6,7-dihydroxy-isochroman	2.20	1.82	1.41	2.12	0.37	0.006
	pos_6111	Urocanic acid	1.78	2.02	1.63	1.74	0.23	0.047
	pos_1499	O-Methylcorypalline	1.58	1.03	1.81	1.89	0.44	0.012
	pos_6083	1-Pyrroline	1.95	1.44	2.13	2.32	0.41	0.008
	pos_1733	(+)-2,3-Dihydro-3-methyl-1H-pyrrole	3.31	2.84	3.18	3.54	0.34	0.014
Organic oxygen compounds	neg_2057	(x)-1,2-Propanediol 1-O-b-D-glucopyranoside	1.86	1.51	1.79	1.87	0.16	0.003
	pos_5144	O-Desmethylvenlafaxine glucuronide	1.49	1.15	1.94	1.62	0.45	0.049
	neg_4496	Melibiose	1.18	1.42	1.57	1.86	0.35	0.022
Phenylpropanoids and polyketides	neg_5756	Naringenin 5-rhamnoside	1.06	1.23	1.83	0.94	0.49	0.025

	neg_6434	Rutin	1.11	1.83	1.13	0.87	0.43	0.006
	neg_5944	Taxifolin 3-arabinoside	1.85	2.26	2.49	2.13	0.40	0.071
Benzenoids	neg_4947	5-(10,13-Nonadecadienyl)-1,3-benzenediol	0.39	1.15	0.94	0.76	0.37	0.014
	neg_6359	Homoveratric acid	3.10	3.44	3.83	3.32	0.62	0.025
Nucleosides, nucleotides, and analogues	neg_1997	5-Fluorouridine	1.29	1.79	1.87	1.68	0.20	0.000
Hydrocarbon derivatives	neg_5720	(R)-11,12,13-Trinor-1(5),6,9-guaiatrien-8-one	0.57	1.41	1.57	1.01	0.49	0.010
Unclassified	pos_4965	8-Isoquinoline methanamine (hydrochloride)	2.03	1.31	2.24	2.35	0.48	0.006
	neg_2213	N-Acetyl-DL-tryptophan	0.47	2.03	2.21	1.22	0.88	0.010
	pos_2575	1alpha,25-dihydroxy-11alpha-[(1R)-oxiranyl]vitamin D3	1.56	2.29	1.85	1.79	0.31	0.005
	pos_4196	PE(17:1(9Z)/0:0)	1.96	2.25	2.05	1.84	0.17	0.005
	neg_4539	Prostaglandin D1 Alcohol	1.10	1.37	1.66	1.58	0.30	0.018
	pos_5973	B-D-Glucopyranosiduronic acid	1.03	1.59	2.01	1.15	0.48	0.008

Fig. S1 Multy samples Rarefaction Curves.

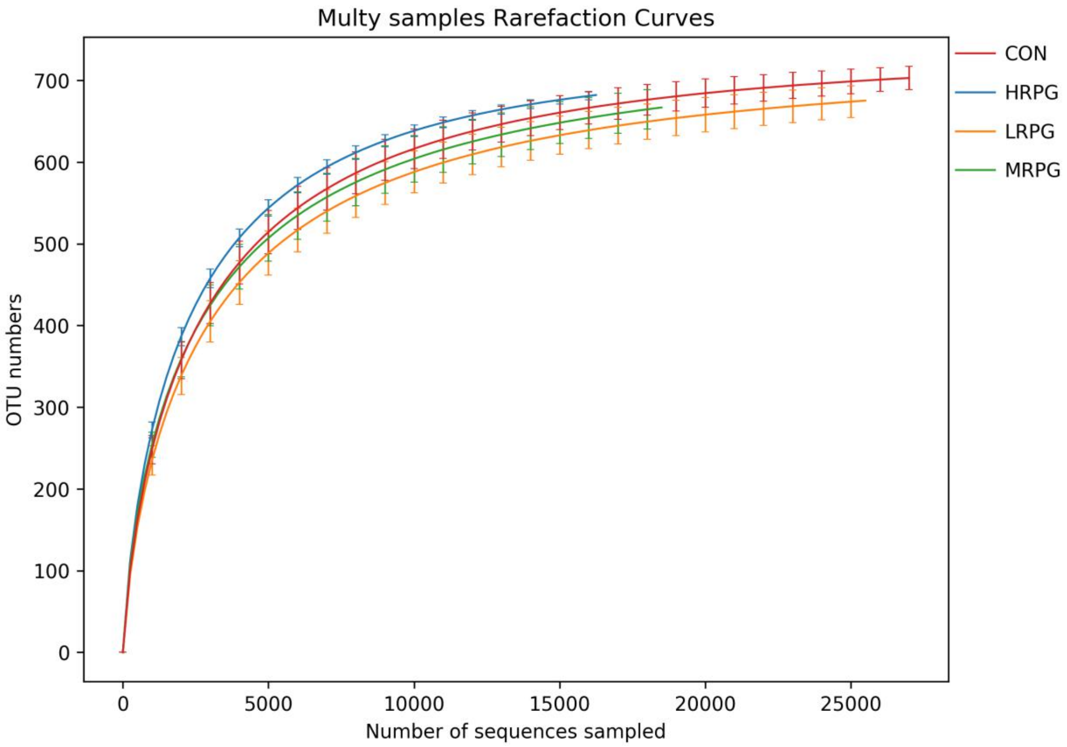

Fig. S2 Venn diagram demonstrates overlap of bacterial OTUs at 97% identity level for CON, LRPG, MRPG, HRPG group. CON, control group, a basal diet; LRPG, low RPG, a basal diet plus 200 g/d RPG; MRPG, medium RPG, a basal diet plus 350 g/d RPG; HRPG, high RPG, a basal diet plus 500 g/d RPG.

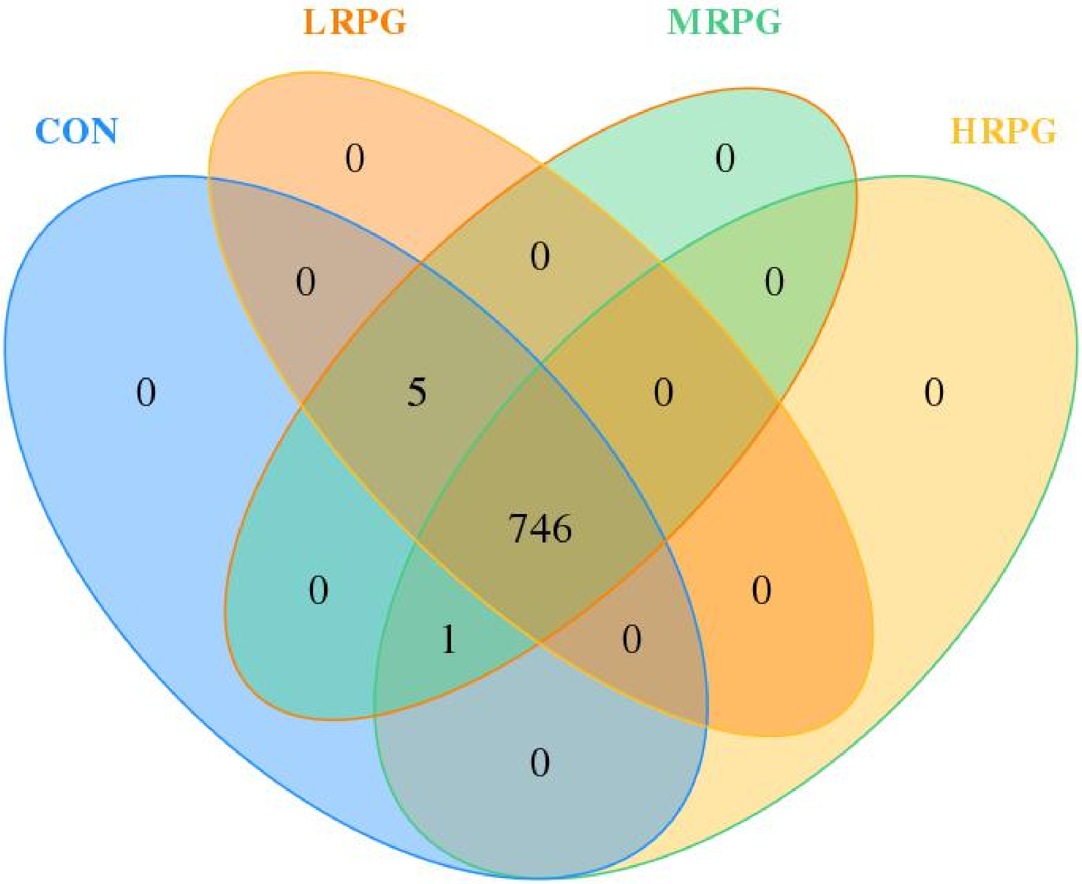

Fig. S3 The HMDB Compound Classification of 315 identified metabolites.

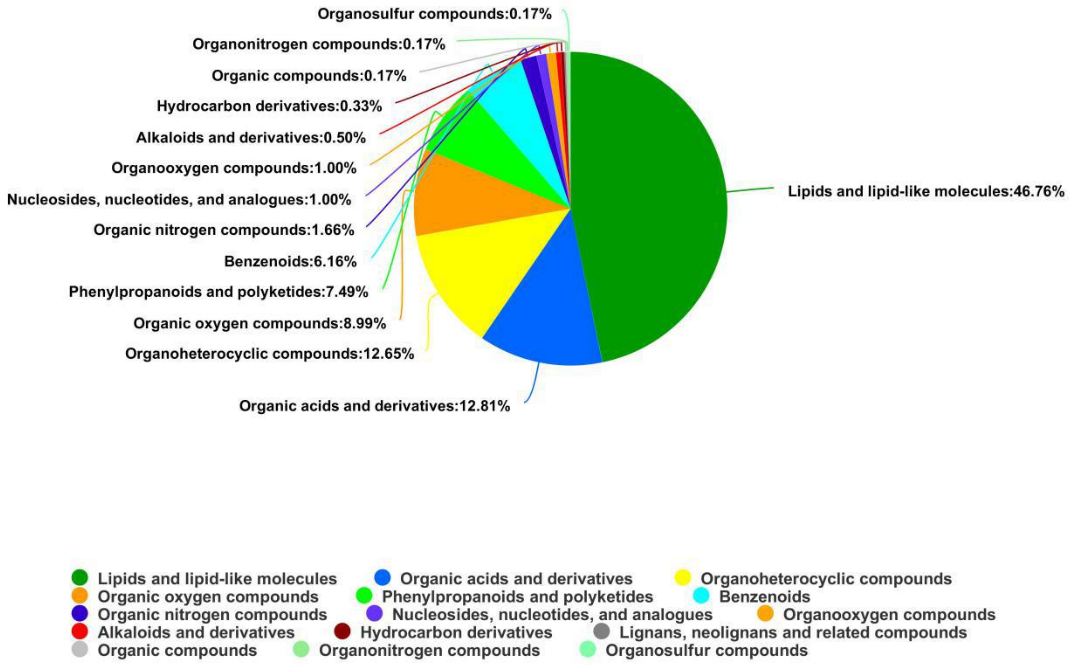

Fig. S4 Total PCA plot of the ruminal fluid samples corresponding to different diets.

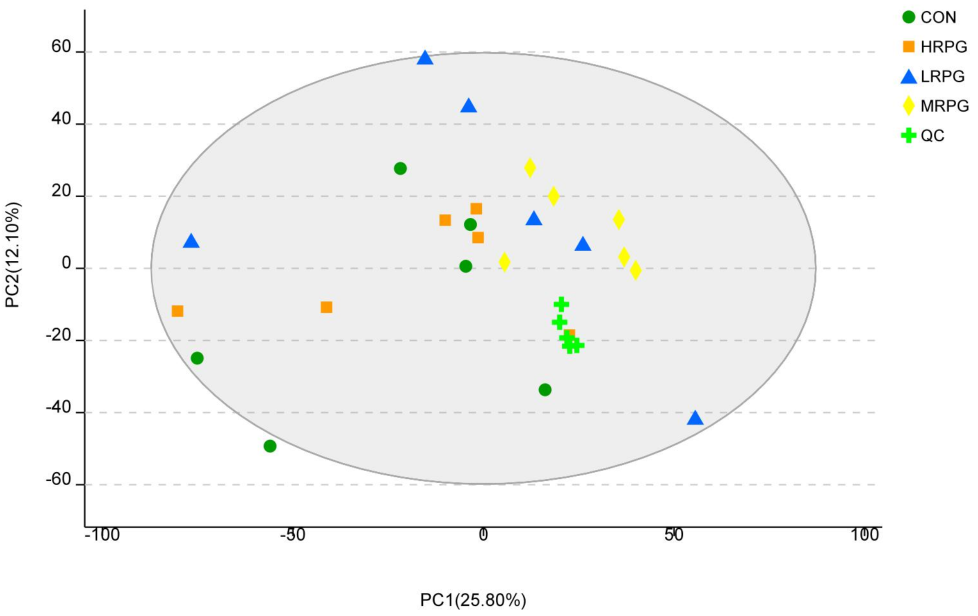

Fig. S5 OPLS-DA score plots and corresponding validation plots derived from the LC-MS metabolite profiles of rumen samples for cows fed increasing doses of RPG supplementary in their diets.

a

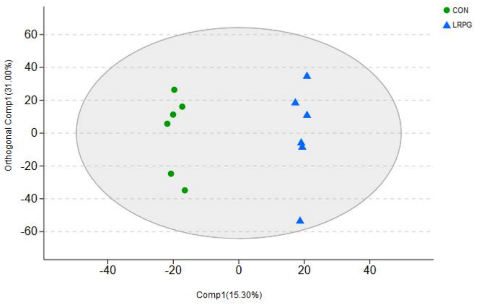

b

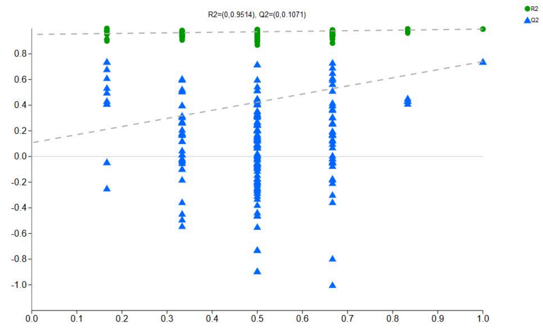

c

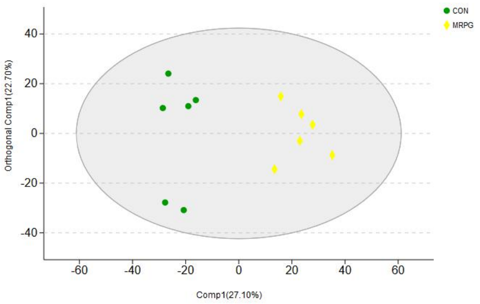

d

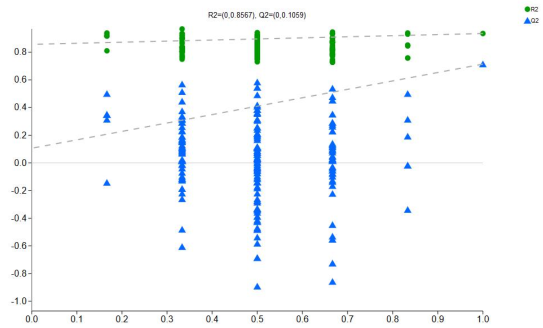

e

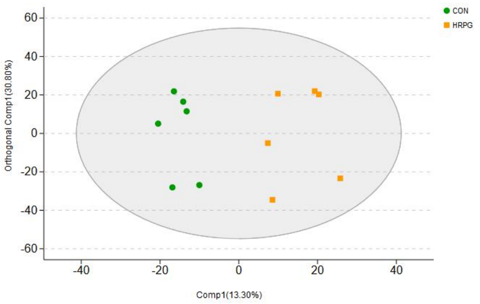

f

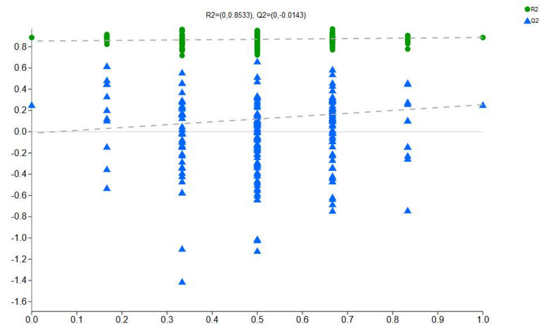

g

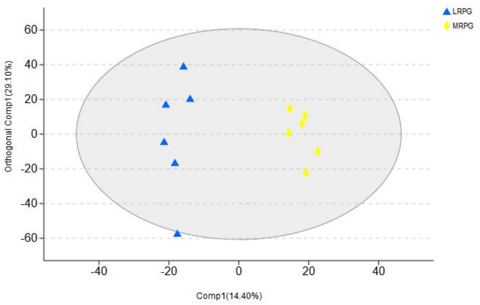

h

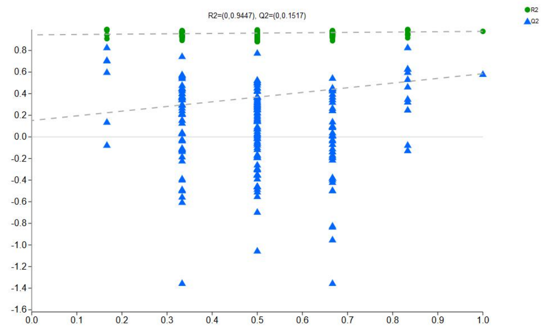

i

j

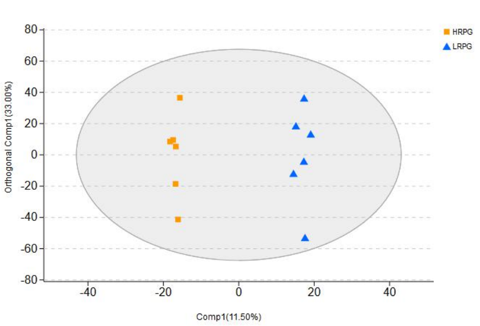
HRPG
LRPG
Orthogonal Comp1(33.00%)
Comp1(11.50%)

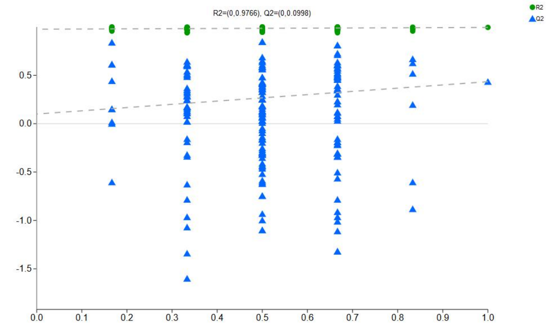
R2=(0,0.9766), Q2=(0,0.0998)
R2
Q2

k

l

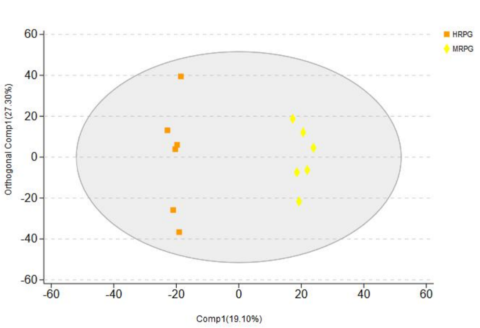
HRPG
MRPG
Orthogonal Comp1(27.30%)
Comp1(19.10%)

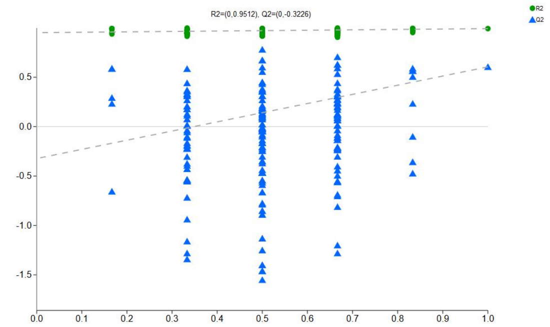
R2=(0,0.9512), Q2=(0,-0.3226)
R2
Q2

Fig. S6 The HMDB Compound Classification of 80 identified differential metabolites.

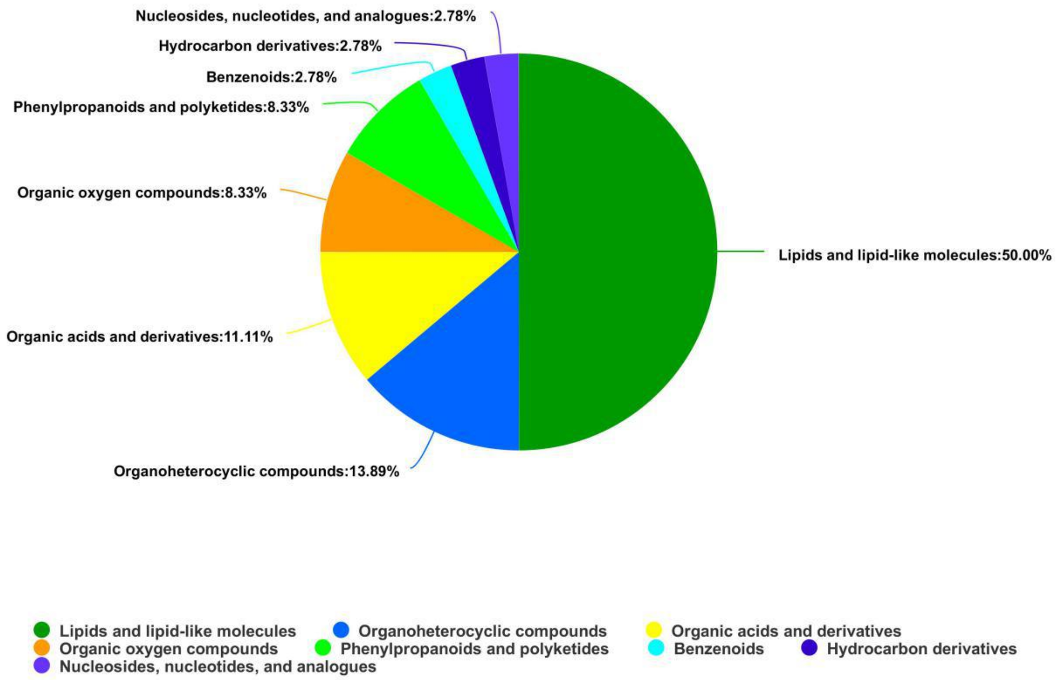

Supplement: Supplemental file 1 [file AEM.01908-20-s0001.pdf]
